# Repair of subtotal tympanic membrane perforations: A temporal bone study of several tympanoplasty materials

**DOI:** 10.1371/journal.pone.0222728

**Published:** 2019-09-19

**Authors:** Mostafa M. A. S. Eldaebes, Thomas G. Landry, Manohar L. Bance

**Affiliations:** Department of Surgery, Division of Otolaryngology, Nova Scotia Health Authority, Halifax, Nova Scotia, Canada; University of New South Wales, AUSTRALIA

## Abstract

The aim of this project was to investigate the effects of different types of graft material, and different remaining segments of the native TM on its motion. In twelve human temporal bones, controlled TM perforations were made to simulate three different conditions. (1) Central perforation leaving both annular and umbo rims of native TM. (2) Central perforation leaving only a malleal rim of native TM. (3) Central perforation leaving only an annular rim of native TM. Five different graft materials (1) perichondrium (2) silastic (3) thin cartilage (4) thick cartilage (5) Lotriderm^®^ cream were used to reconstruct each perforation condition. Umbo and stapes vibrations to acoustic stimuli from 250 to 6349 Hz were measured using a scanning laser Doppler vibrometer. Results showed that at low frequencies: in the Two Rims condition, all grafting materials except thick cartilage and Lotriderm cream showed no significant difference in umbo velocity from the Normal TM, while only Lotriderm cream showed a significant decrease in stapes velocity; in the Malleal Rim condition, all materials showed a significant decrease in both umbo and stapes velocities; in the Annular Rim condition, all grafting materials except Lotriderm and perichondrium showed no significant difference from the Normal TM in stapes velocity. Umbo data might not be reliable in some conditions because of coverage by the graft. At middle and high frequencies: all materials showed a significant difference from the Normal TM in both umbo and stapes velocities for all perforation conditions except in the Annular Rim condition, in which silastic and perichondrium showed no significant difference from the Normal TM at umbo velocity in the middle frequencies. In the low frequencies, the choice of repair material does not seem to have a large effect on sound transfer. Our data also suggests that the annular rim could be important for low frequency sound transfer.

## Introduction

The normal tympanic membrane (TM), with its very thin (~100 μm) and complex 3-layered structure, provides both mechanical stability against atmospheric pressure changes and acoustic sensitivity for sound transmission [1]. Perforation of the TM is a common condition encountered in otologic practice, which can be caused by several factors and can lead to a conductive hearing loss up to approximately 50 dB [2]. Tympanoplasty is a common otologic surgery that aims to reconstruct the perforated TM. Since the introduction of tympanoplasty surgery in 1952, several graft materials have been used to reconstruct the perforated TM [3–5]. The unique and complex structure of the TM makes it difficult to find an ideal grafting material that provides both excellent acoustic transmission and the rigid mechanical properties needed for long term stability in diseased ears. Each grafting material seems to have its merits and demerits. Despite the widespread use and high success rate of temporalis fascia, recurrent perforation and retraction of the graft due to negative middle ear pressure are major disadvantages [6]. Cartilage offers more strength and resistance to negative middle ear pressure (and less recurrence of disease because of this) but it can compromise the acoustic quality of transmission due to its increased mass and stiffness [7–9]. Clinical studies investigating different graft materials have shown contradictory results [4,10–14]. However, in clinical studies it’s difficult to control for all the confounding variables. Some experimental studies have studied the acoustic properties of cartilage and other materials that could be used in tympanoplasty. Zahnert et al. [7] studied the acoustic and mechanical characteristics of cartilage of different thicknesses in an artificial ear canal. They found that by reducing the cartilage thickness, the mechanical energy transfer loss could be reduced. Murbe et al. [15] used an ear canal model to investigate the effect of the size of the cartilage transplants and the technique used in reconstructing the TM on sound transmission. They concluded that cutting the thick cartilage into thin plates improves sound transfer properties. They also concluded that the surgical technique used in reconstructing the TM influences sound transmission. Aarnisalo et al. [8,16] studied the effect of the cartilage size and its position using laser holography in a temporal bone study with onlay onto intact TM. They report that although the TM motion is reduced over the grafted TM above 4 kHz, the cartilage grafts did not seem to have a significant effect on stapes motion. Allardyce et al. [17] studied the mechanical properties of silk membranes of different thicknesses alongside cartilage and paper. They suggested that silk membranes might have good hearing outcomes besides providing mechanical support in reconstructing the TM. In a study of the effect of perforations on middle ear sound transmission, Voss et al. [18] found that perforations can cause a frequency dependent loss, which increased as perforation size increased, but it did not depend on perforation location. In a previous temporal bone study by our group we investigated the effect of cartilage overlay on TM and stapes motion. We concluded that at low frequencies, the annular rim seems to be important for acoustic transfer function [9]. To our knowledge, the effect of restructuring a perforated TM with different materials has never been studied before in intact temporal bone samples, which allows much greater control over the experimental variables compared to clinical studies. In this temporal bone study, controlled TM perforations were performed, and different graft materials were created to investigate 1) the effect of different graft materials on TM and stapes motion, and 2) the effect of TM boundaries at the annulus or malleus being covered or uncovered by the graft on TM and stapes motion in tympanoplasty using laser Doppler vibrometry (LDV).

## Materials and methods

This study was reviewed and approved by the Health Sciences Research Ethics Board, Dalhousie University, Nova Scotia, Canada (REB #: 2001–314).

### Temporal bone preparation

Twelve human temporal bones, including the soft tissue and pinna, were obtained from the Anatomy Gifts Registry (Hannover, MD). Informed consent was obtained from donor's legal next-of-kin, or the donor themselves, in all cases according to Anatomy Gifts Registry. The bones were harvested from donors within 24–48 hours after death and were kept frozen until thawed. All donors had no reported otologic disease. All temporal bones were examined microscopically to exclude any otologic pathology. A phase difference of 180 degrees at low frequencies between the oval and round window was checked in each temporal bone to make sure that there were no air bubbles inside the cochlea that could have been created during the freezing and thawing process [19]. Cortical mastoidectomy with extended posterior tympanotomy was performed to expose the stapes. The external auditory canal was drilled down to about 2mm from the TM annulus. A brass ring with two holes drilled in the side of it was glued to the remnant edge of the ear canal using coral glue (Eco Tech Marine LLC, Allentown, PA). A 1mm burr was used to drill a hole in the posterior wall of the middle ear cavity to allow for the insertion of a middle ear microphone tube. Reflective microbeads weighing less than 0.05 mg (3M, Minneapolis, MN) were applied over the stapes posterior crus and footplate to enhance laser beam reflection. Normal saline was applied to the TM periodically throughout testing and any excess fluid was removed by suction [20]. To keep the temporal bones hydrated and to delay putrefaction, the bones were refrigerated in 5°C in approximately 300 cc of normal saline with 10 ml of 10% Betadine during overnight periods for around 3–5 nights. A more detailed description of the temporal bone preparation, including a photograph of the experimental setup, is given in a previous publication [9].

### Measurements

**Fig 1:**
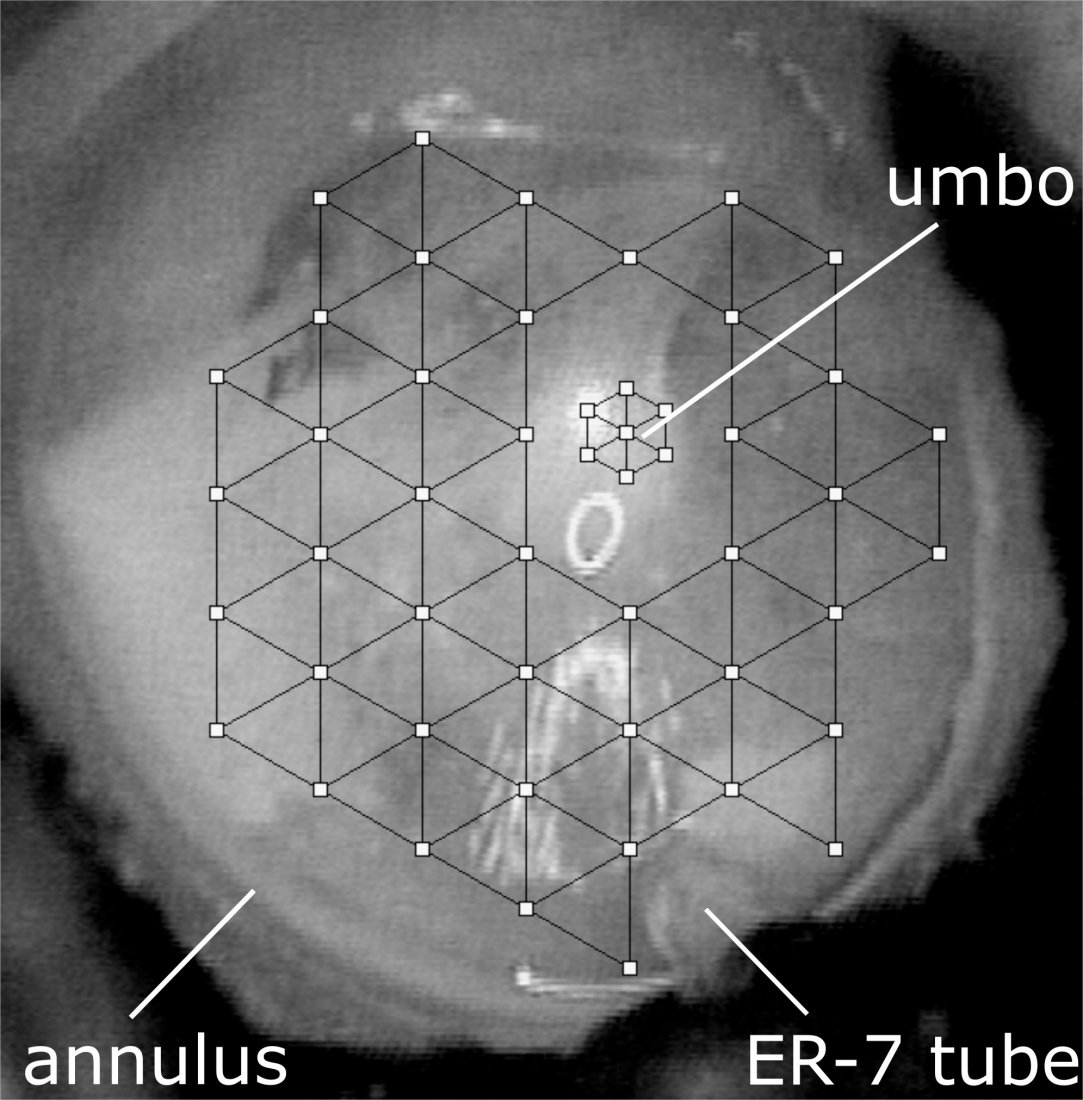
The LDV measurement grid used for a central perforation leaving an annular rim condition repaired by covering with perichondrium. A larger coarser grid with equally spaced points was created for TM measurement to measure this large surface, and a similar but smaller grid was created for the umbo measurement more accurately.

The same measurement system and data analysis methods from our previous study were used [9], and are described in more detail in that publication. Pure tones from 250 to 6349Hz in 1/3 octave steps at 110 dB SPL (re: 20 μPa) was presented to an ER-3A sound delivery tube tip (Etymotic Research, Elk Grove Village, IL, USA), which was placed through a hole in the brass ring approximately 5 mm from the umbo. The ear canal sound pressure level (SPL) (P_ec_) was monitored using an ER-7 probe tube microphone (Etymotic Research) inserted through a second hole in the brass ring with the probe tip placed within 2 mm of the TM. In this study, middle ear cavity pressure (P_mec_) measurements were collected using an Etymotic Research ER-7 probe tube microphone inserted through a hole in the posterior wall of the middle ear cavity with its tip placed close to the promontory. To maintain the ear canal pressure during measurements, a glass coverslip was placed over the brass ring and sealed with petroleum jelly. TM, umbo and stapes vibrations were measured using a Polytec (Tustin, CA) OFV-5000 laser Doppler vibrometer. TM vibrations were collected from about 40–50 equally spaced points in a triangular grid pattern. A separate smaller grid of 7 points was created to collect measurements from the umbo for averaging (Fig 1).

Stapes vibration measurements were collected in separate trials with identical stimuli by directing the LDV laser beam at the reflective beads that were placed over the stapes footplate.

A data acquisition device (DAQ NI USB-6259, National Instruments, Austin, TX, USA) was used to record the vibrometer output and produce the sound stimulus signal. Noise floor amplitude during sound trials was measured on the bony promontory. Vibration measurements less than 5 dB above noise were rejected.

### TM grafts preparation

The different graft materials were prepared as follows:

Cartilage grafts: Full thickness cartilage pieces from the cavum conchae and the tragus were obtained from the tissue specimen. Zahnert et al. [7] showed no significant difference in sound transfer characteristics between conchal and tragal cartilage. After removing the perichondrium, cartilage pieces were cut into 0.5 mm (thin cartilage) and 1.0 mm (thick cartilage) grafts using a commercially available cartilage-cutting device (Kurz Company, Dusslingen, Germany). Cartilage pieces were then cut using a sharp scalpel into small pieces to be used as TM grafts for the different TM conditions. Thickness of the cartilage grafts was measured using high-resolution ultrasonography (Vevo 2100 system; Fujifilm VisualSonics, Toronto, Canada) to confirm its thickness.

Perichondrium: Perichondrium was harvested from both tragal and conchal cartilages. Any skin or subcutaneous tissue remnants were removed off the perichondrium bluntly. The graft was then left on a plate to dry out before use.

Silastic grafts: A 0.1 mm silicone sheet (Medtronic Xomed, Jacksonville, FL USA) was cut into small pieces using a size 10-scalpel blade.

Lotriderm cream: Approximately 0.1 ml of Lotriderm cream (Schering Plough) applied through a syringe was used to create a sheet of cream to graft the perforated TM.

**Fig 2:**
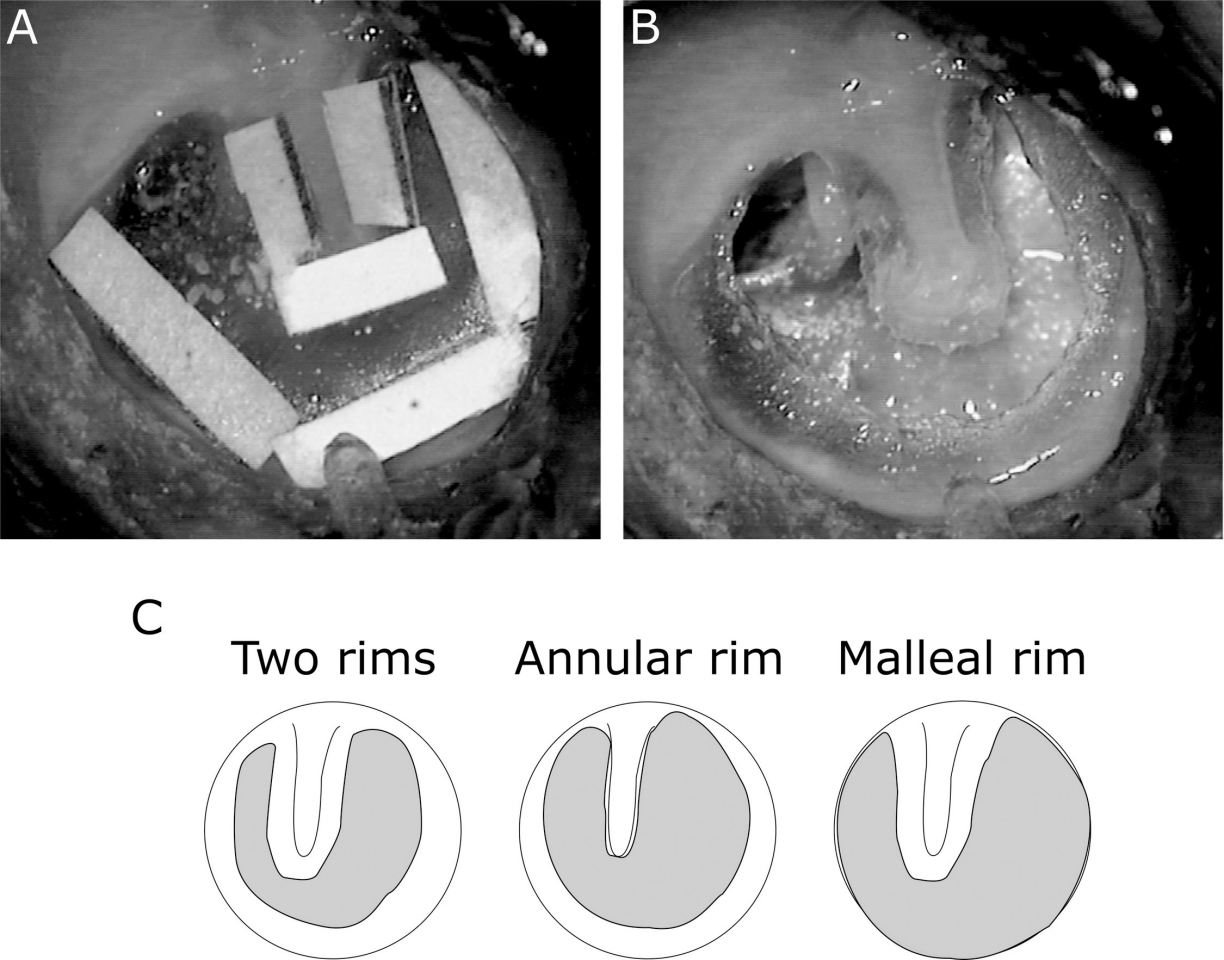
TM before and after perforation. (A) TM with 1 mm paper patches applied above the TM boundaries to highlight the TM remnants that we kept for various conditions, i.e. the patch next to the umbo was kept for malleus rim conditions and the patch next to the annulus for annular rim conditions. (B) TM with central perforation leaving two rims to show how the TM actually looked for the annular rim and malleus rim strips. (C) A drawing to show different perforated rim conditions, with the grey indicating the TM region removed.

**Fig 3:**
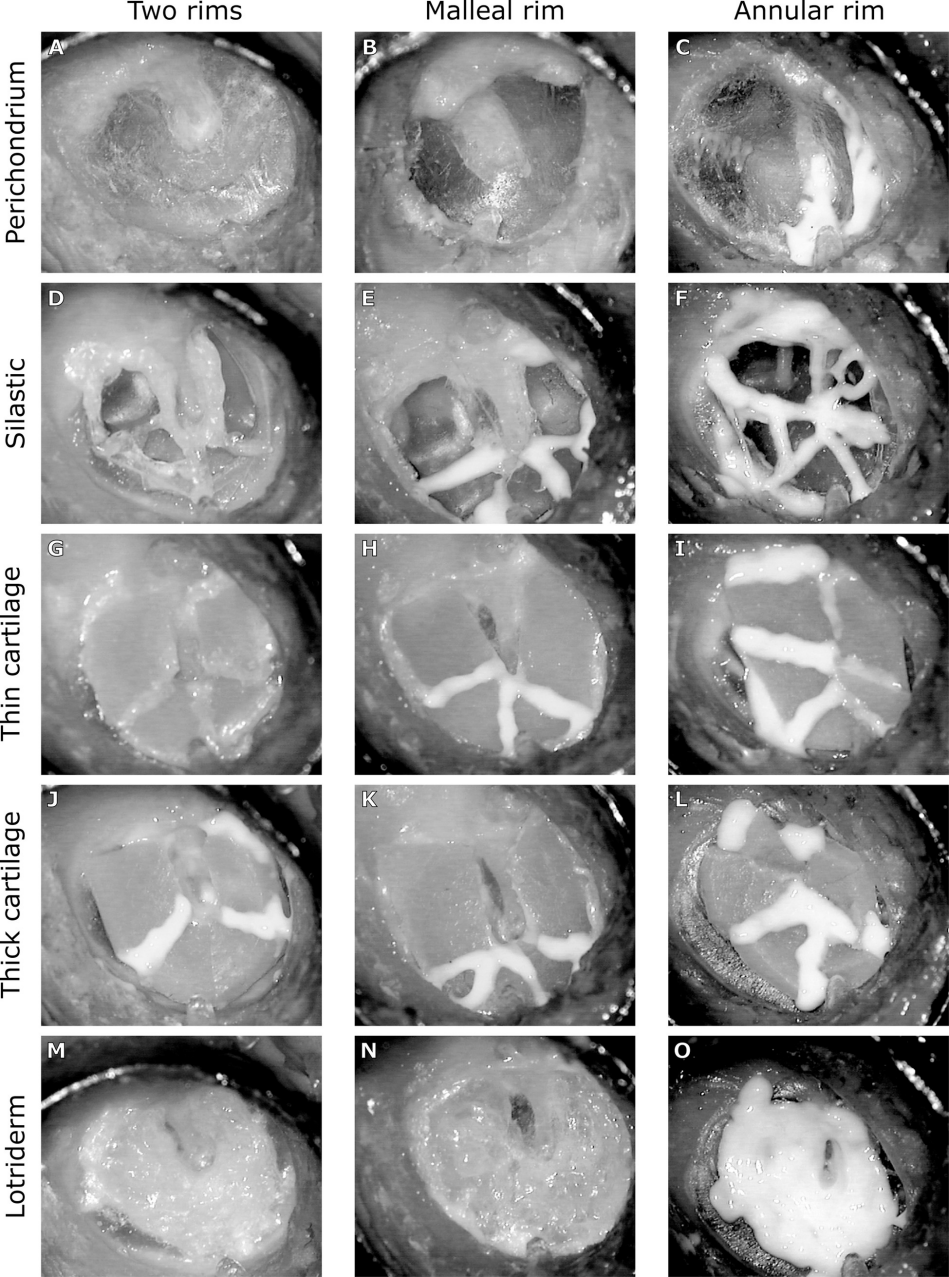
Examples of the different grafts in the three rim conditions (columns). The two rims column represents central perforation leaving two rims (i.e. both an annular rim and a malleus rim (similar to Fig 2B), with the hole covered by different graft materials. The malleal rim column represents central perforation leaving malleal rim intact but with no annular rim, covered by different graft materials. The annular rim column represents central perforation leaving annular rim intact but with no malleal rim, covered by different graft materials. Rows(A-C) represents perichondrium graft. Rows (D-F) represents silastic graft. Rows (G-I) represents thin cartilage. Rows (J-L) represents thick cartilage. Rows (M-O) represents Lotriderm cream alone. In all cases any gaps between graft pieces were sealed with Lotriderm cream.

After taking baseline measurements from the normal TM, pieces of paper (approximately 1mm width) were cut and placed over the TM boundaries, the area of the TM not covered by the paper patches were cut using a needle to produce a central perforation leaving two free rims of the TM, one around the umbo and one around the annulus (Two Rims [2R] condition; Fig 2B), as TM boundaries have been claimed to be important for flexible membrane vibrations [9,21]. Different TM grafts were then used to close the perforation, producing five different TM grafting conditions: 1- Perichondrium. 2- Silastic. 3- Thin cartilage. 4- Thick cartilage. 5- Lotriderm cream (Fig 3).

The different edges of the different graft pieces used to reconstruct the TM were placed close together without any duplication over the edges of the TM remnants. Only in the annular rim (AR) conditions there might be overlay/duplication of the graft materials over the umbo. Any small air gaps between the graft edges or between the graft and the TM were always patched with ~0.03 ml of Lotriderm cream to ensure a sealed “ear canal”. A complete seal at the TM, as well as at the brass ring/coverslip, was confirmed by a large recorded difference in dB SPL between the ear canal and middle ear to acoustic stimuli. TM and stapes measurements were collected after applying each grafting material for the Two Rims condition. Next, one of the TM boundaries was removed, leaving either the malleal rim (MR) (Figs 2C and 3) or annular rim (AR) intact (Figs 2C and 3). The five different graft materials were then used to close the perforation and measurements were collected. Twelve temporal bones were harvested in total. Of these, ten bones were perforated as described for the 2R condition. After recording the 2R condition, the perforation was extended to give either the MR condition (5 bones) or the AR condition (5 bones). In two additional bones, the 2R condition recording was not done, with only the MR (1 bone) or AR (1 bone) condition recorded. Therefore, the pool of data collected before data analysis for the 2R, MR, and AR was 10, 6, and 6 respectively. During data analysis, one of the temporal bones (a bone that was 2R/MR) showed a floppy TM segment and was excluded from our pool of data. The final group n’s for 2R, MR, and AR conditions were 9, 5, and 6, respectively.

### Data analysis: Umbo and stapes

A similar data analysis protocol from our previous study was used [9]. Analysis of the different umbo and stapes measured points was performed using software written in LabVIEW (National Instruments, Austin, TX, USA). The vibration velocities were normalized to pressure differential across the TM (PΔTM = P_ec_—P_mec_) and converted to dB re: 1 mm/s/Pa. Only points that showed agreement in amplitude across frequencies (i.e. similar response curve as other points) were averaged into a single value per condition/frequency per bone, as the lack of agreement with other umbo or stapes points indicated an unstable measurement point.

**Fig 4:**
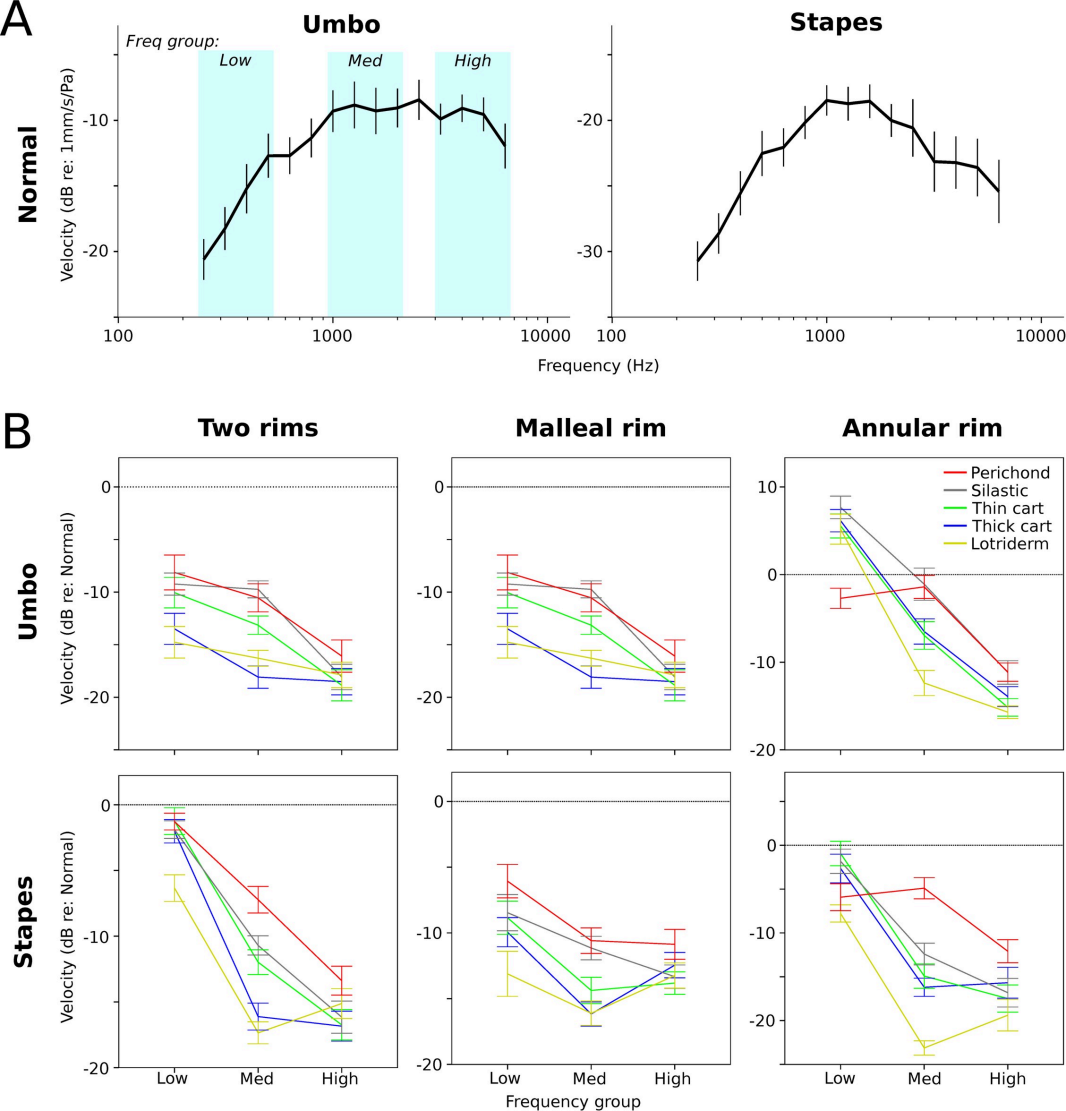
Mean velocities for the Normal TM and the different graft materials. A) Umbo and stapes vibration velocity for the Normal TM. The three frequency group ranges are also illustrated. B) Umbo and stapes velocity means relative to the Normal TM for the different graft materials under the three rim conditions. Data are shown for the low frequency range, mid-frequency range, and high frequency range. In general, patterns for umbo and stapes velocity are similar in all conditions. Leaving an annular rim (two rims and annular rim) seems to help preserve the low frequency responses. Stiffer and high mass reconstructions seem to have worse high frequency responses. The data shown was that used for statistical analyses. Error bars = ±1 SEM.

Both the umbo and the stapes data were analyzed statistically using SPSS version 24.0 (IBM Corp., Armonk, NY, USA). During the assessment, frequency responses were pooled into three primary classifications of frequencies: low frequency group (250–500 Hz), middle frequency group (1000–2000 Hz), and high frequency group (3174–6349 Hz). Within these three frequency groups, the umbo and stapes vibration velocities for the Normal condition were verified to be normally distributed (Shapiro-Wilk test, *p* > 0.05). The Normal mean velocities are shown in (Fig 4A).

For each frequency, values for graft conditions were then normalized to the Normal bone condition. Type I error rate for statistical testing was lowered to 0.05/3 = 0.0167 because the data were split into the three frequency groups. In each frequency grouping, a one-way analysis of variance (ANOVA) was used to check for statistically significant differences between the different conditions across the frequency groups. The same frequency-grouped ANOVA method was also done comparing the means of the three rim conditions, pooled across graft conditions. Because velocities were made relative to the Normal condition within bones, it was not possible to include the Normal condition in the ANOVAs, as all Normal values became zero.

In order to compare graft conditions to Normal, each graft/frequency group/rim combination was tested for statistical difference from zero using a one-sample *t* test, providing a *p* value for differences from Normal. The 5 grafts, 3 frequency groups, and 3 rim conditions, gives a total of 45 *t* tests. Therefore, the type I error rate was reduced to 0.05/45 = 0.00111 for these tests.

### Data analysis: TM

**Fig 5:**
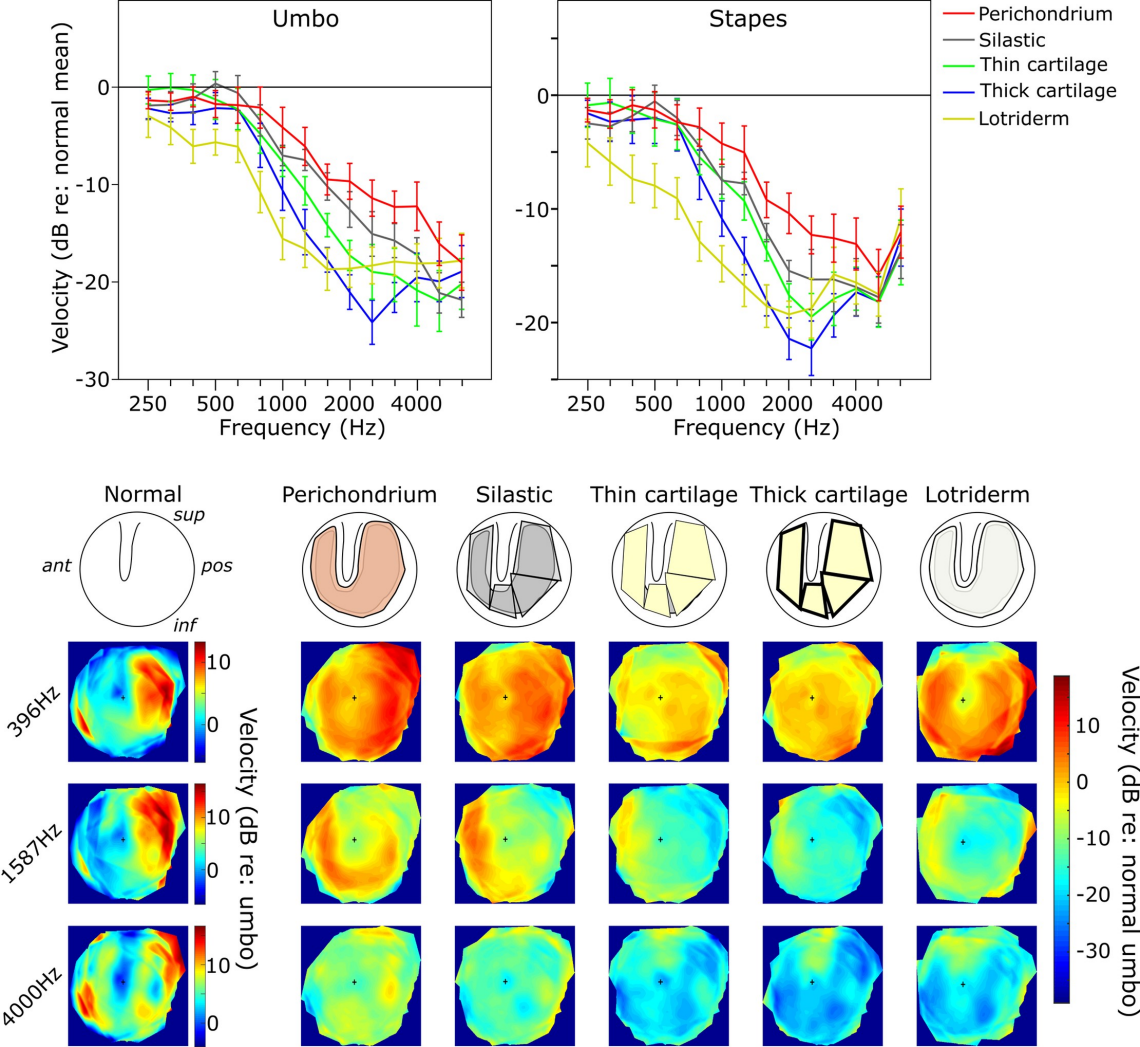
TM map showing central perforation leaving Two Rims condition. Upper line plots show the mean umbo and stapes amplitudes relative to the Normal TM responses across frequencies. Error bars = ±1 SEM. Most reconstructions are reasonable in restoring lower frequencies with both rims present, but in the higher frequencies, there appears to be a graded effect with larger mass/stiffer reconstructions having worse high frequency responses. The lower images show the TM amplitude maps averaged across bones. This shows the patterns of vibrations on the TM for different reconstruction materials. The Normal data are scaled relative to the Normal umbo value, whereas the Two Rims condition maps are shown relative to Normal across the TM by subtracting the averaged Normal map from each averaged overlay map. The small black cross in each map indicates the umbo position.

**Fig 6:**
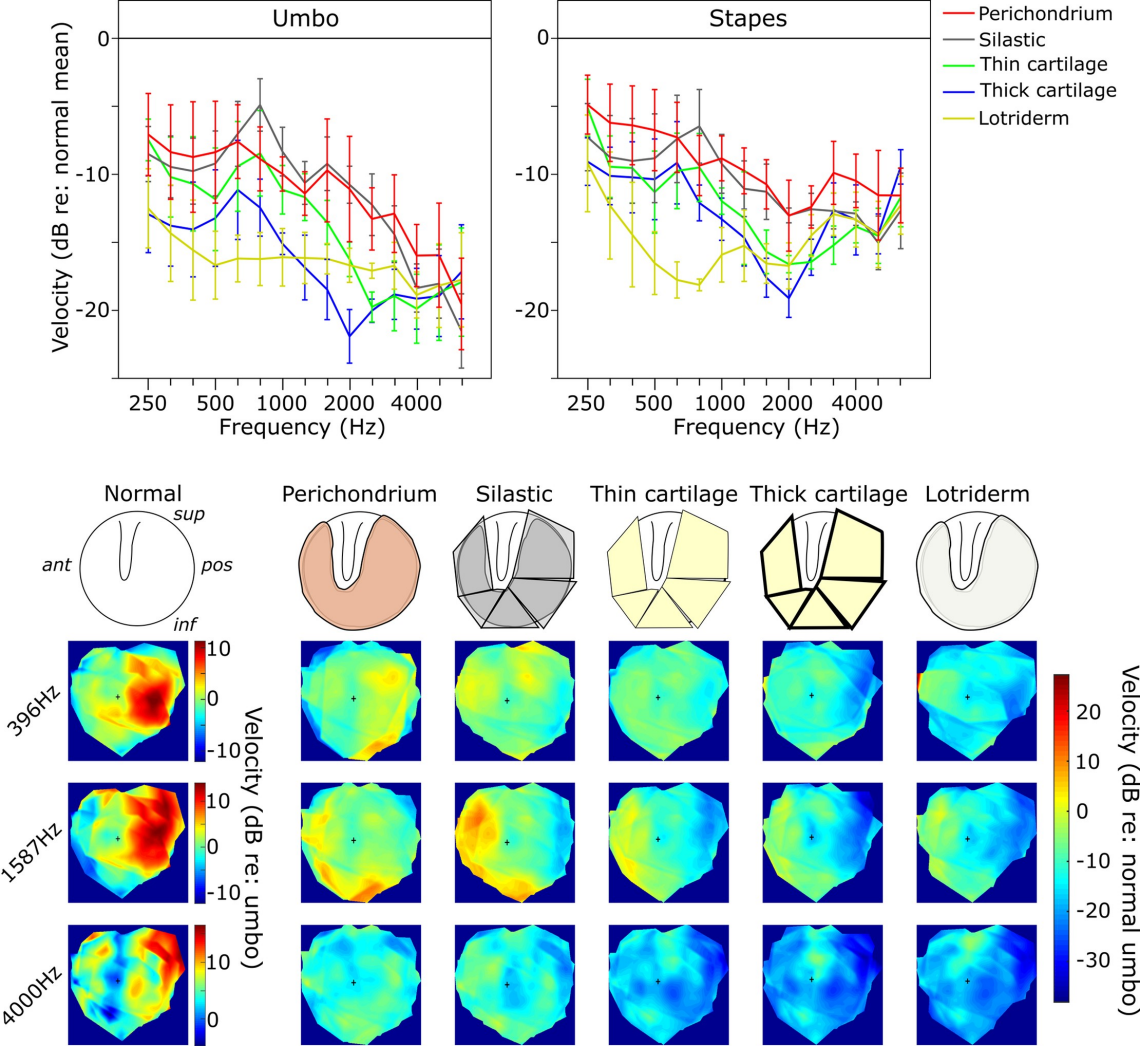
TM map showing central perforation leaving just the Malleal Rim condition. Upper line plots show the mean umbo and stapes amplitudes relative to Normal across frequencies. None of the reconstructions seem able to restore the low frequency responses in this condition. In the higher frequencies, again there is a suggestion of decreasing responses with increasing mass/stiffness of the reconstruction. Error bars = ±1 SEM. The lower images show the TM amplitude maps averaged across bones. The Normal data are scaled relative to the Normal umbo value, whereas the Malleal Rim condition maps are shown relative to Normal across the TM by subtracting the averaged Normal map from each averaged overlay map. The small black cross in each map indicates the umbo position.

**Fig 7:**
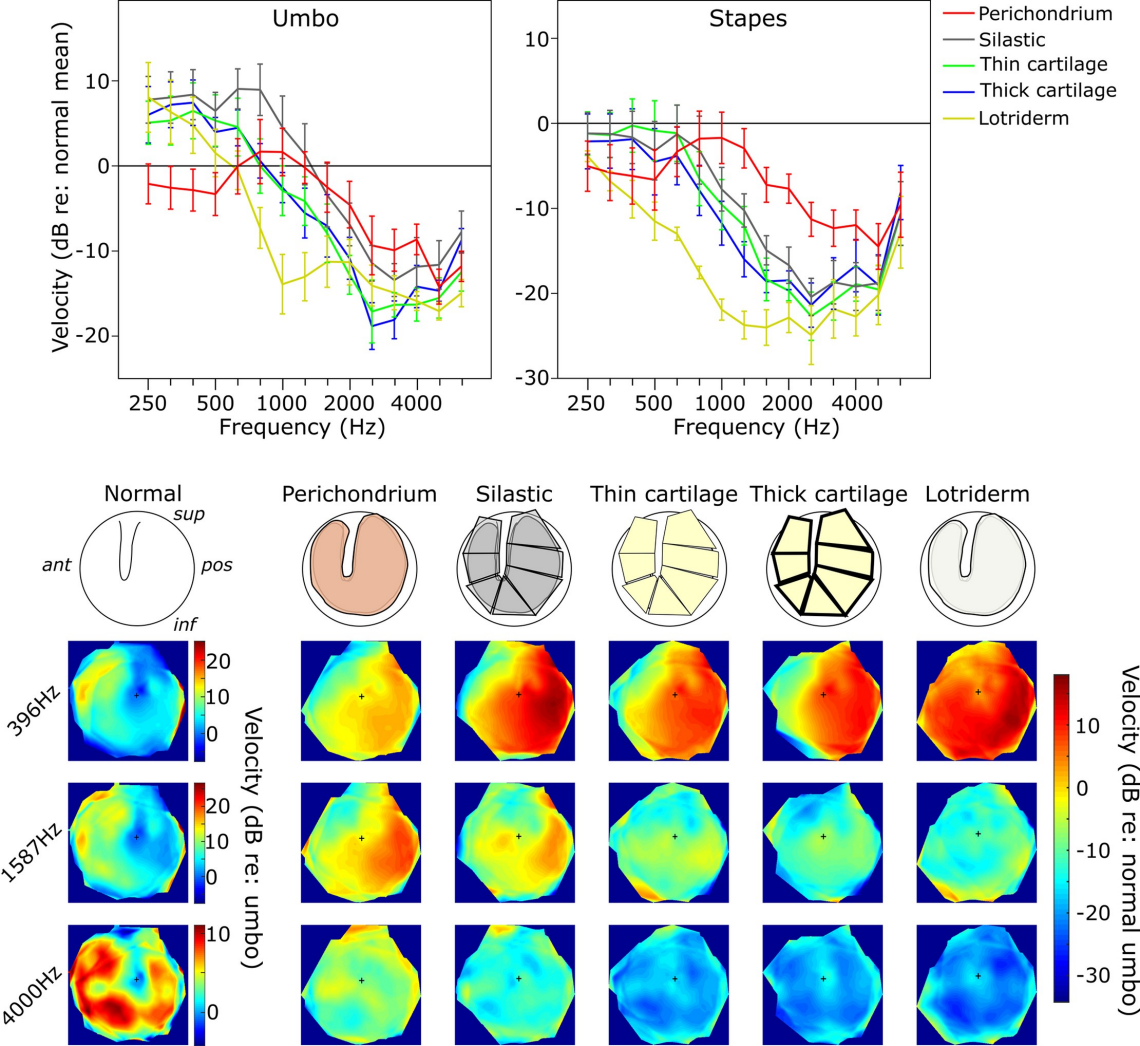
TM map showing central perforation leaving just the Annular Rim condition. Upper line plots show the mean umbo and stapes amplitudes relative to Normal across frequencies. Error bars = ±1 SEM. In this condition, it seems that most reconstructions can restore low frequency responses to a close to normal level. The lower images show the TM amplitude maps averaged across bones to show vibration patterns across the TM. The Normal data are scaled relative to the Normal umbo value, whereas the Annular Rim condition maps are shown relative to Normal across the TM by subtracting the averaged Normal map from each averaged overlay map. The small black cross in each map indicates the umbo position.

For qualitative analysis, further data processing was done for the grid of measurements across the TM surface. Points that exhibited amplitude or phase responses that deviated greatly from neighboring points at low frequencies (<2000Hz) were removed for all further analyses, including at high frequencies, as a valid measurement point should move similarly to adjacent points at low frequencies [22]. The grid coordinates for remaining points were co-registered across conditions in each bone by centering the umbo positions, aligning the orientation of the malleus handle, and stretching the point coordinates according to the TM bony rim height and width for each condition to correct for differences in TM position relative to the LDV scanning head between graft/rim conditions. For any right ears (n = 5), the coordinates were flipped horizontally to orient them as left ears. All grid values within bones were made relative (dB difference) to the Normal condition umbo value for each frequency and each condition. Using Matlab (Mathworks, Natick, MA, USA), interpolated color maps of the velocities were generated for each bone/condition for different stimulus frequencies with the “griddata” Matlab function. For each overlay condition, the interpolated map pixel data for the Normal condition was then subtracted from the condition pixel data. Consequently, the resulting color maps demonstrate how the vibration amplitudes across the TM-graft structure differed from Normal. Then at the umbo position, these difference maps were aligned across all bones and then averaged. These averaged difference plots are shown at three selected frequencies in Figs 5–7 for each condition, plus the averaged color maps for the Normal condition, plotted as dB re: umbo.

## Results

The results are sectioned by rim condition and recording site (umbo or stapes), and further sub-sectioned by frequency group. While the following section lists the findings in text, a better appreciation of the overall effects of graft and TM condition on the results is garnered by examination of the figure and table numbers given at the beginning of each subsection.

### Two rims condition, umbo velocity

See S1 Table and Fig 5.

**Low frequencies:** Thick cartilage and Lotriderm conditions showed a significant difference from the Normal TM condition with a mean magnitude of -2.4 and -4.7 dB respectively. Lotriderm also showed a significant difference from all other grafting materials at low frequencies except thick cartilage. Perichondrium, silastic and thin cartilage showed no significant difference from the Normal TM condition or from each other.

**Middle frequencies:** All grafting materials showed a significant difference from the Normal TM condition by means of -7.3, -9.3, -12.4, -16.1 and -17.4 dB for perichondrium, silastic, thin cartilage, thick cartilage and Lotriderm respectively. Thick cartilage showed no significant difference either from thin cartilage or from Lotriderm. Silastic showed neither significant difference from perichondrium nor from thin cartilage. All other grafting materials showed significant differences between each other.

**High frequencies:** All grafting materials showed a significant difference from the Normal TM condition by means of -14.7, -19.0, -20.6, -20.0 and -18.0 dB for perichondrium, silastic, thin cartilage, thick cartilage and Lotriderm respectively. Only perichondrium showed a significant difference from thin and thick cartilage.

### Two rims condition, stapes velocity

See S2 Table and Fig 5.

**Low frequencies:** Only the Lotriderm showed a significant difference from the Normal TM with a mean of -6.3 dB. Lotriderm also showed a significant difference from all other grafting materials. Perichondrium, silastic, thin and thick cartilage showed no significant difference either from the Normal TM condition or from each other.

**Middle frequencies**: All materials showed a significant decrease in stapes velocity from the Normal TM by a mean of -7.2, -10.7, -12.0, -16.1and -17.4 dB for perichondrium, silastic, thin cartilage, thick cartilage and Lotriderm respectively. There were a number of significant differences between graft materials as shown in S2 Table.

**High frequencies:** All materials showed a significant decrease in stapes velocity from the Normal TM by a mean magnitude of -13.4, -16.2, -16.7, - 16.8 and -15.1 dB for perichondrium, silastic, thin cartilage, thick cartilage and Lotriderm respectively. There was no significant difference between any of the graft materials in the high frequencies.

### Malleal rim condition, umbo velocity

See S3 Table and Fig 6.

There was a significant decrease in umbo velocity from the Normal TM condition with all the graft materials used across the different frequency groups. The different grafting materials did not show any significant difference between each other in both umbo and stapes velocities at both low and high frequencies except for Lotriderm and perichondrium, which showed a significant difference only at low frequencies.

**Low frequencies:** The significant differences from Normal were -8.1, -9.2, -10.1, -13.5 and -14.8 dB for perichondrium, silastic, thin cartilage, thick cartilage and Lotriderm respectively. Only the perichondrium and Lotriderm conditions showed a significant difference between each other.

**Middle frequencies:** The significant differences from Normal were -10.5, -9.7, -13.1, - 18.1 and -16.3 dB for perichondrium, silastic, thin cartilage, thick cartilage and Lotriderm respectively. Thick cartilage velocity was significantly lower than all other materials except Lotriderm, whereas Lotriderm was less than all except both thick and thin cartilage.

**High frequencies:** The significant differences from Normal were -16.1, -18.1, -18.9, - 18.5 and -17.9 dB for perichondrium, silastic, thin cartilage, thick cartilage and Lotriderm respectively.

### Malleal rim condition, stapes velocity

See S4 Table and Fig 6.

There was a significant decrease in stapes velocity from the Normal TM condition with the all the graft materials used across the different frequency groups.

**Low frequencies:** The significant differences from Normal were -6.1, -8.5, -8.8, -9.9 and -13.1 dB for perichondrium, silastic, thin cartilage, thick cartilage and Lotriderm respectively. Only the perichondrium and Lotriderm conditions showed a significant difference between each other.

**Middle frequencies:** The significant differences from Normal were -10.6, -11.1, -14.4, -16.2 and -16.1 dB for perichondrium, silastic, thin cartilage, thick cartilage and Lotriderm respectively.

**High frequencies**: The significant differences from Normal were -10.9, -13.3, -13.9, - 12.5 and -13.2 dB for perichondrium, silastic, thin cartilage, thick cartilage and Lotriderm respectively.

### Annular rim condition, umbo velocity

See S5 Table and Fig 7.

It should be noted that the umbo velocity measurements for the Annular Rim condition might not be trustworthy as the different grafting materials were covering the umbo area of the TM-graft structure. Therefore, the vibrations measured were those of the graft materials rather than the umbo, and there may not have been perfect mechanical coupling of the two.

**Low frequencies:** All the grafting materials showed a significant increase in umbo velocity from the Normal TM except for the perichondrium. Perichondrium was significantly lower than all other graft materials. The differences from Normal were -2.8, +7.7, +5.6, +6.2 and +5.2 dB for perichondrium, silastic, thin cartilage, thick cartilage and Lotriderm respectively.

**Middle frequencies:** Both silastic and perichondrium showed no significant difference from the Normal TM. All other grafting materials showed a significant difference. Lotriderm was significantly lower than silastic and perichondrium. The differences from Normal were -1.4, -1.1, -7.0, -6.5 and -12.4 dB for perichondrium, silastic, thin cartilage, thick cartilage and Lotriderm respectively.

**High frequencies:** All grafting materials showed a significant difference from the Normal TM condition. The differences from Normal were -11.1, -11.2, -15.2, -13.9 and -15.8 dB for perichondrium, silastic, thin cartilage, thick cartilage and Lotriderm respectively.

### Annular rim condition, stapes velocity

See S6 Table and Fig 7.

**Low frequencies:** There was a significant decrease in stapes velocity from the Normal TM condition with only Lotriderm and perichondrium. All other grafting materials showed no significant difference. The differences from Normal were -5.9, -1.8, -0.9, -2.7 and -7.8 dB for perichondrium, silastic, thin cartilage, thick cartilage and Lotriderm respectively.

**Middle frequencies:** All materials showed a significant decrease in stapes velocity from the Normal TM by means of -4.9, -12.4, -14.9, -16.2 and -23.1 dB for perichondrium, silastic, thin cartilage, thick cartilage and Lotriderm respectively. All grafting materials showed a significant difference between each other except for thin, thick cartilage and silastic. Perichondrium was significantly better than all other grafting materials.

**High frequencies:** All materials showed a significant decrease in stapes velocity from the Normal TM by means -12.1, -16.8, -17.5, -15.7 and -19.4 dB for perichondrium, silastic, thin cartilage, thick cartilage and Lotriderm respectively. There was no significant difference between any of the graft materials in the high frequencies.

### Comparison of overall perforation effects on umbo and stapes velocity

See Fig 8.

**Fig 8:**
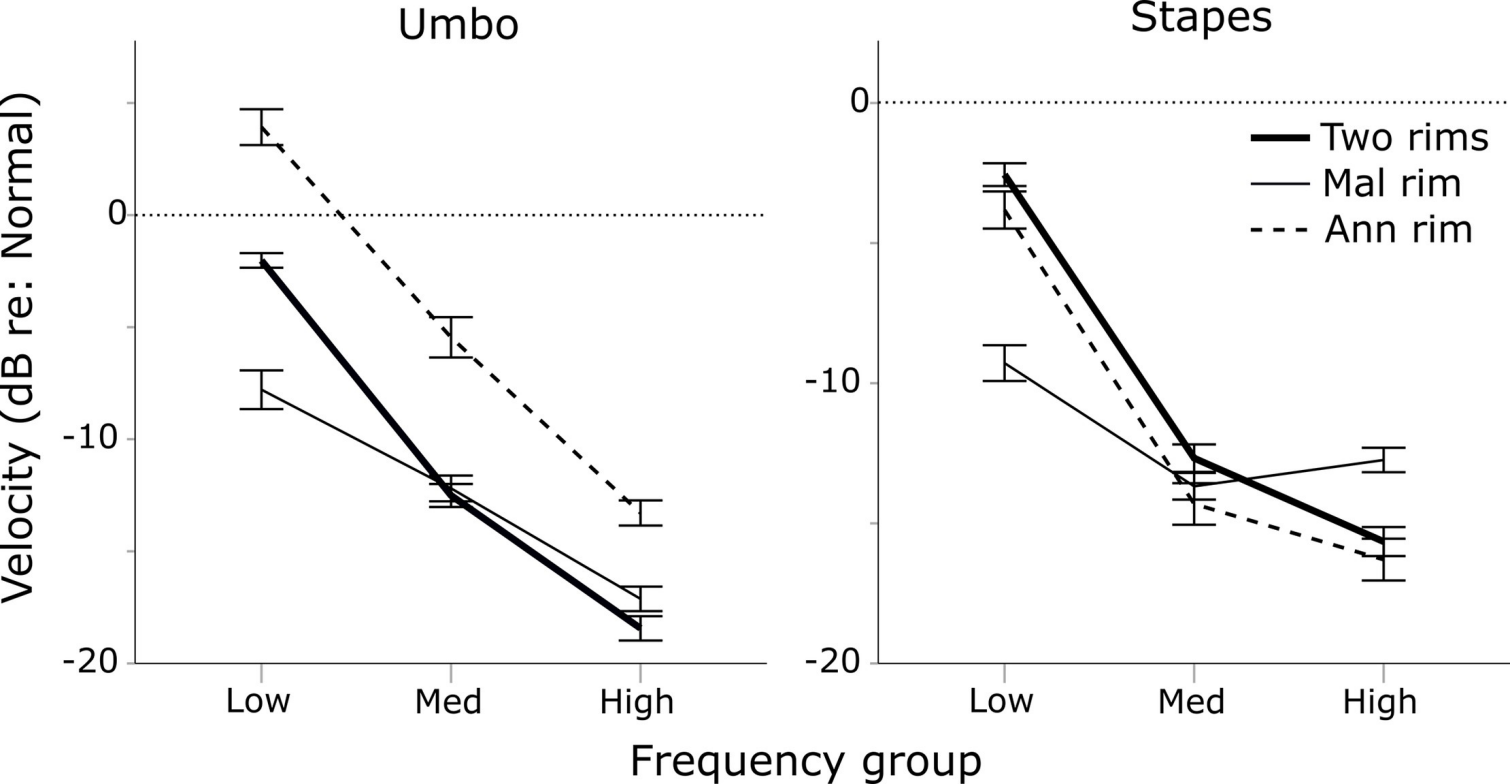
Frequency group means for umbo and stapes velocity relative to Normal TM. Each perforation condition is pooled across graft materials. Error bars ± 1 SEM. The results suggest that conditions where an annular rim is preserved (Two Rims and Annular Rim), low frequency responses are better restored than the condition where this annular rim is not preserved, whereas mid to high frequencies are similarly reduced by any perforation.

**Low frequencies:** For the umbo, there were significant decreases in velocity for the 2R and MR conditions by means of -2.0 and -7.8 dB respectively, whereas AR showed a significant increase of 3.9 dB compared to Normal. Again, AR umbo data may not reflect actual umbo vibrations. The perforation condition means were all significantly different from each other. For the stapes, all perforation types resulted in significantly reduced velocity compared to Normal, with mean losses of -2.6, -9.3, and -3.8 dB for 2R, MR, and AR respectively. MR was significantly lower than both 2R and AR at the stapes.

**Middle frequencies:** At the umbo, significant decreases below Normal were found for all perforation conditions, with means of -12.5, -12.2, and -5.5 dB for 2R, MR, and AR. AR was significantly greater than the other two conditions. For the stapes, significant decreases from Normal were also found, with means of -12.7, -13.7, and -14.3 dB for 2R, MR and AR.

**High frequencies:** For the umbo, all conditions were significantly lower than Normal, with means of -18.4, -17.1, and -13.3 dB for 2R, MR, and AR. AR was significantly greater than the other two rim conditions. At the stapes, all perforation conditions were also significantly below Normal, with means of -15.7, -12.7, and -16.3 dB for 2R, MR and AR. MR was significantly greater than the other two conditions, but only by about 3 dB on average.

The TM amplitude color maps show some interesting observations related to our TM data analysis. The posterior quadrant of the TM seems to vibrate with the most intensity in the Normal TM condition, especially at low frequencies. At mid and high frequencies, the posterior part of the TM seems to be vibrating less than at low frequencies. The posterior part of the TM seems to be important in determining the overall vibration pattern of the umbo. With the reconstructed TM in different conditions, it seems that when the vibration pattern of the posterior quadrant of a condition is dampened, there is an overall amplitude loss of the umbo velocity. Figs 5–7.

## Discussion

One of the main findings in this study is that annular rim of the TM seems to be important for low frequency sound transmission. These findings in the current study agree with findings of our earlier study [9], in which graft materials were overlaid on the intact native TM without perforations.

In the 2R condition, at low frequencies, perichondrium, silastic and thin cartilage showed no significant difference from the Normal TM for umbo velocities, while for stapes velocities all grafting materials showed no significant difference from the Normal TM except Lotriderm. In the mid and high frequencies, all of the grafting materials showed a significant difference. In other words, with both rims intact any graft material except Lotriderm can restore low frequency transmission, but none can restore high frequency transmission, and the level of middle frequency restoration is related to graft mass and/or stiffness. Being a thick fluid, Lotriderm has a completely different material property than all the other graft materials tested here, which is likely related to its failure to restore low frequency transmission. Creams can be used to repair the perforation in the outpatient clinic setting to gain some appreciation of how much gain might be expected from TM repair, and so we were interested in comparing results with cream to results with actual surgical materials.

When the AR of the TM was removed leaving only the MR intact, at low frequencies there was a significant reduction in both umbo and stapes velocities in all grafting materials used. The greatest reduction was about 14 and 13 dB for both umbo and stapes respectively with using Lotriderm cream as a graft. At mid and high frequencies all grafting materials showed a significant drop in both umbo and stapes velocities.

If the MR was removed, leaving the AR intact, silastic, thin cartilage and thick cartilage showed no significant difference from the Normal TM at low frequencies for stapes velocities. This seems to highlight the importance of the AR for low frequency function. Both Lotriderm and perichondrium showed a significant reduction from the Normal TM, but in terms of actual dB loss they were very similar to the other graft materials. It is possible that the baffle effect (i.e. the ability of perichondrium and Lotriderm to block sound and create a transmembrane pressure differential, which will affect low frequency function) with large perforations is not as good as it is for more rigid materials. On the other hand, perichondrium stood out as significantly outperforming all other graft materials for mid frequencies when the AR was intact. As mentioned in the Results section, the umbo recordings for the AR condition may not be trustworthy as graft materials often covered the umbo, so those results are not discussed here.

The explanation for the apparent significance of the AR at low frequencies could have several possible causes, also discussed in our previous study [9]. For example, the annular area of the TM has a flexible and larger surface area than the relatively less flexible conical area close to the umbo. By losing the flexible outer area of the TM, the TM impedance could be increased relative to the air impedance [23,24].

The sound pressure difference across the TM is considered the primary drive for TM and ossicular vibration. TM perforation-induced loss results mainly from change in the pressure difference across the TM [18].

In interpreting the findings in our study, it seems that at low frequencies (when TM moves in phase) [22], it does not matter as much which material is used to reconstruct the TM as long as it can restore the pressure difference across the TM. While at mid and high frequencies it seems the mass effect of the material used (or possibly stiffness, because in our materials the mass and stiffness co-varied) starts to be a factor. By looking at the order of grafting materials in Figs 4–7, perichondrium seems to affect the mid to high frequencies the least, followed by silastic, thin cartilage and thick cartilage. The more flexible and less mass the material is, the less there is a drop in the mid to high frequencies, especially in the middle frequencies.

Lotriderm is not as solid as other grafting materials used in our experiment and hence it might allow some pressure to pass through the perforated TM. This could be the explanation for the significant difference that we see between Lotriderm and other materials. It should be noted that there are some characteristics for the Normal TM that is difficult to replicate ex-vivo such as the tension of the TM and its attachment to the ossicular chain. This could explain why perichondrium repair motion in our results was significantly lower than the Normal TM at low frequencies for the AR condition.

Although some studies found that thinner cartilage grafts <0.5mm had better acoustic transfer properties than a 1mm cartilage graft [7,25], in our study there was not much difference between both.

It is important to acknowledge a few possible shortcomings in our study within which our results need to be interpreted carefully. One of the possible shortcomings is that the stapes motion measurement angle wasn’t corrected for as the measurement angle, which was likely 30–45 degrees, and would have reduced responses by a couple of dB, and might have an effect on the stapes velocity measurements [26], but in general our measurement angle was relatively constant between conditions and since we are reporting relative changes in amplitude, these relative measurements are still valid. Also, there are some differences between our cadaveric set up and clinical applications, for instance perichondrium stayed dry in our preparations, but would have been wetted by body moisture or blood in surgical conditions. Of course, we cannot replicate changes in graft materials with healing, scarring and fibrosis.

## Conclusion

Our findings in this study on human cadaver temporal bones suggest that the annular rim seems to be important for low frequency vibration transmission, whereas middle and high frequencies were similarly reduced by all perforation conditions. Also, it seems that at low frequencies it does not matter which material is used to reconstruct the TM, as there were not significant differences in our study between the grafting materials at low frequencies except Lotriderm, whereas for the middle frequencies, the material mass made a significant difference, with lighter materials (especially perichondrium) giving the best performance. High frequencies were similarly impaired with all graft materials.

## Supporting information

S1 Table Umbo velocities for central perforation leaving Two Rims condition. Summary of the significant differences between the different grafting materials and the normal TM umbo velocities for central perforation leaving Two Rims condition
* = the mean difference is significant at the .0167 level for comparisons between graft conditions, and 0.00111 for graft-Normal comparisons.
(DOCX)

S2 Table Stapes velocities for central perforation leaving Two Rims condition. Summary of the significant differences between the different grafting materials and the normal TM stapes velocities for central perforation leaving Two Rims condition
* = the mean difference is significant at the .0167 level for comparisons between graft conditions, and 0.00111 for graft-Normal comparisons.
(DOCX)

S3 Table Umbo velocities for central perforation leaving Malleal Rim condition. Summary of the significant differences between the different grafting materials and the normal TM umbo velocities for central perforation leaving Malleal Rim condition
* = the mean difference is significant at the .0167 level for comparisons between graft conditions, and 0.00111 for graft-Normal comparisons
N.B. Umbo results for the Malleal Rim might not be reliable as the grafting materials covered the umbo.
(DOCX)

S4 Table Stapes velocities for central perforation leaving Malleal Rim condition. Summary of the significant differences between the different grafting materials and the normal TM stapes velocities for central perforation leaving Malleal Rim condition
* = the mean difference is significant at the .0167 level for comparisons between graft conditions, and 0.00111 for graft-Normal comparisons.
(DOCX)

S5 Table Umbo velocities for central perforation leaving Annular Rim condition. Summary of the significant differences between the different grafting materials and the normal TM umbo velocities for central perforation leaving Annular Rim condition
* = the mean difference is significant at the .0167 level for comparisons between graft conditions, and 0.00111 for graft-Normal comparisons.
(DOCX)

S6 Table Stapes velocities for central perforation leaving Annular Rim condition. Summary of the significant differences between the different grafting materials and the normal TM stapes velocities for central perforation leaving Annular Rim condition
* = the mean difference is significant at the .0167 level for comparisons between graft conditions, and 0.00111 for graft-Normal comparisons.
(DOCX)
