# Supplementary material for: Repair of subtotal tympanic membrane perforations: A temporal bone study of several tympanoplasty materials
Source: PLoS One. 2019 Sep 19;14(9):e0222728. doi: 10.1371/journal.pone.0222728 (PMC6752791; doi:10.1371/journal.pone.0222728)
Supplement: S1 Table — Summary of the significant differences between the different grafting materials and the normal TM umbo velocities for central perforation leaving Two Rims condition * = the mean difference is significant at the .0167 level for comparisons between graft conditions, and 0.00111 for graft-Normal comparisons. (DOCX) [file pone.0222728.s001.docx]

**S1 Table.** Summary of the significant differences between the different grafting materials and the normal TM umbo velocities for ***central perforation leaving Two Rims condition***

*= the mean difference is significant at the .0167 level for comparisons between graft conditions, and 0.00111 for graft-Normal comparisons.

| UMBO velocity | | Low Freq (250-500)  Mean dB difference (SE) | Middle Freq (1000-2000)  Mean dB difference (SE) | High Freq (3174-6349) Mean dB difference (SE) |
| --- | --- | --- | --- | --- |
| normal | thickCart | -2.406 (.587) *  *p* < 0.0005 | -16.064 (1.113) *  *p* < 0.0005 | -20.001 (1.082) *  *p* < 0.0005 |
| normal | thinCart | -.477 (.785) | -12.436 (.919) *  *p* < 0.0005 | -20.572 (1.474) *  *p* < 0.0005 |
| normal | silastic | -1.126 (.681) | -9.315 (.758) *  *p* < 0.0005 | -18.974 (.980) *  *p* < 0.0005 |
| normal | Lotriderm | -4.710 (.877) *  *p* < 0.0005 | -17.374 (1.001) *  *p* < 0.0005 | -17.970 (1.082) *  *p* < 0.0005 |
| normal | perichond | -1.394 (.508) | -7.342 (.976) *  *p* < 0.0005 | -14.656 (1.194) *  *p* < 0.0005 |
| thickCart | thinCart | -1.929 (.990) | -3.627 (1.358) | .571 (1.661) |
| thickCart | silastic | -1.280 (.990) | -6.748 (1.358) *  *p* < 0.0005 | -1.027 (1.661) |
| thickCart | Lotriderm | 2.304 (.990) | 1.310 (1.358) | -2.030 (1.661) |
| thickCart | perichond | -1.012 (.990) | -8.721 (1.358) *  *p* < 0.0005 | -5.345 (1.661) *  *p* =0.015 |
| thinCart | silastic | .649 (.990) | -3.120 (1.358) | -1.598 (1.661) |
| thinCart | Lotriderm | 4.233 (.990) *  *p* < 0.0005 | 4.937 (1.358) *  *p* =0.004 | -2.602 (1.661) |
| thinCart | perichond | .917 (.990) | -5.093 (1.358) *  *p* =0.002 | -5.916 (1.661) *  *p* =0.005 |
| silastic | Lotriderm | 3.584 (.990) *  *p* =0.004 | 8.058 (1.358) *  *p* < 0.0005 | -1.003 (1.661) |
| silastic | perichond | .268 (.990) | -1.972 (1.358) | -4.318 (1.661) |
| Lotriderm | perichond | -3.316 (.990) *  *p* =0.01 | -10.031 (1.358) *  *p* < 0.0005 | -3.314 (1.661) |
