# Supplementary material for: Repair of subtotal tympanic membrane perforations: A temporal bone study of several tympanoplasty materials
Source: PLoS One. 2019 Sep 19;14(9):e0222728. doi: 10.1371/journal.pone.0222728 (PMC6752791; doi:10.1371/journal.pone.0222728)
Supplement: S2 Table — Summary of the significant differences between the different grafting materials and the normal TM stapes velocities for central perforation leaving Two Rims condition * = the mean difference is significant at the .0167 level for comparisons between graft conditions, and 0.00111 for graft-Normal comparisons. (DOCX) [file pone.0222728.s002.docx]

**S2 Table.** Summary of the significant differences between the different grafting materials and the normal TM stapes velocities for ***central perforation leaving Two Rims condition***

*= the mean difference is significant at the .0167 level for comparisons between graft conditions, and 0.00111 for graft-Normal comparisons.

| STAPES velocity | | Low Freq (250-500)  Mean dB difference (SE) | Middle Freq (1000-2000)  Mean dB difference (SE) | High Freq (3174-6349) Mean dB difference (SE) |
| --- | --- | --- | --- | --- |
| normal | thickCart | -2.019 (.887) | -16.117 (1.022) *  *p* < 0.0005 | -16.843 (1.136) *  *p* < 0.0005 |
| normal | thinCart | -1.243 (1.019) | -11.979 (.943) *  *p* < 0.0005 | -16.746 (1.158) *  *p* < 0.0005 |
| normal | silastic | -1.893 (.665) | -10.703 (.729) *  *p* < 0.0005 | -16.156 (1.122) *  *p* < 0.0005 |
| normal | Lotriderm | -6.342 (1.012) *  *p* < 0.0005 | -17.351 (.838) *  *p* < 0.0005 | -15.124 (1.139) *  *p* < 0.0005 |
| normal | perichond | -1.282 (.636) | -7.223 (1.008) *  *p* < 0.0005 | -13.383 (1.097) *  *p* < 0.0005 |
| thickCart | thinCart | -.776 (1.216) | -4.137 (1.294)*  *p* =0.016 | -.0971 |
| thickCart | silastic | -.125 (1.216) | -5.413 (1.294) *  *p* < 0.0005 | -0.686 |
| thickCart | Lotriderm | 4.322 (1.216) *  *p* =0.005 | 1.234 (1.294) | -1.718 |
| thickCart | perichond | -.737 (1.216) | -8.893 (1.294) *  *p* < 0.0005 | -3.459 |
| thinCart | silastic | .650 (1.216) | -1.275 (1.294) | -.589 |
| thinCart | Lotriderm | 5.098 (1.216) *  *p* < 0.0005 | 5.372 (1.294) *  *p* =0.001 | -1.621 |
| thinCart | perichond | 0.386 (1.216) | -4.755 (1.294) *  *p* =0.003 | -3.362 |
| silastic | Lotriderm | 4.448 (1.216) *  *p* =0.003 | 6.647 (1.294) *  *p* < 0.0005 | -1.032 |
| silastic | perichond | -.611 (1.216) * | -3.479 (1.294) | -2.773 |
| Lotriderm | perichond | -5.060 (1.216) *  *p* < 0.0005 | -10.127 (1.294) *  *p* < 0.0005 | -1.740 |
