# Supplementary material for: Repair of subtotal tympanic membrane perforations: A temporal bone study of several tympanoplasty materials
Source: PLoS One. 2019 Sep 19;14(9):e0222728. doi: 10.1371/journal.pone.0222728 (PMC6752791; doi:10.1371/journal.pone.0222728)
Supplement: S3 Table — Summary of the significant differences between the different grafting materials and the normal TM umbo velocities for central perforation leaving Malleal Rim condition * = the mean difference is significant at the .0167 level for comparisons between graft conditions, and 0.00111 for graft-Normal comparisons N.B. Umbo results for the Malleal Rim might not be reliable as the grafting materials covered the umbo. (DOCX) [file pone.0222728.s003.docx]

**S3 Table.** Summary of the significant differences between the different grafting materials and the normal TM umbo velocities for ***central perforation leaving Malleal Rim condition***

| UMBO velocity | | Low Freq (250-500)  Mean dB difference (SE) | Middle Freq (1000-2000)  Mean dB difference (SE) | High Freq (3174-6349)  Mean dB difference (SE) |
| --- | --- | --- | --- | --- |
| normal | thickCart | -13.496 (1.481) *  *p* < 0.0005 | -18.083 (1.060) *  *p* < 0.0005 | -18.521 (1.252) *  *p* < 0.0005 |
| normal | thinCart | -10.051 (1.447) *  *p* < 0.0005 | -13.148 (.869) *  *p* < 0.0005 | -18.860 (1.480) *  *p* < 0.0005 |
| normal | silastic | -9.234 (1.052) *  *p* < 0.0005 | -9.737 (.808) *  *p* < 0.0005 | -18.076 (1.207) *  *p* < 0.0005 |
| normal | Lotriderm | -14.771 (1.506) *  *p* < 0.0005 | -16.285 (.756) *  *p* < 0.0005 | -17.880 (1.211) *  *p* < 0.0005 |
| normal | perichond | -8.134 (1.655) *  *p* < 0.0005 | -10.545 (1.336) *  *p* < 0.0005 | -16.083 (1.536) *  *p* < 0.0005 |
| thickCart | thinCart | -3.445 (2.040) | -4.935 (1.399) *  *p* =0.006 | .339 (1.902) |
| thickCart | silastic | -4.262 (2.040) | -8.346 (1.399) *  *p* < 0.0005 | -.444 (1.902) |
| thickCart | Lotriderm | 1.275 (2.040) | -1.798 (1.399) | -.640 (1.902) |
| thickCart | perichond | -5.362 (2.040) | -7.538 (1.399) *  *p* < 0.0005 | -2.437 (1.902) |
| thinCart | silastic | -.817 (2.040) | -3.411 (1.399) | -.784 (1.902) |
| thinCart | Lotriderm | 4.720 (2.040) | 3.137 (1.399) | -.980 (1.902) |
| thinCart | perichond | -1.917 (2.040) | -2.603 (1.399) | -2.777 (1.902) |
| silastic | Lotriderm | 5.537 (2.040) | 6.548 (1.399) *  *p* < 0.0005 | -.196 (1.902) |
| silastic | perichond | -1.100 (2.040) | .808 (1.399) | -1.993 (1.902) |
| Lotriderm | perichond | -6.637 (2.040) *  *p* =0.016 | -5.740 (1.399) *  *p* =0.001 | -1.797 (1.902) |

*= the mean difference is significant at the .0167 level for comparisons between graft conditions, and 0.00111 for graft-Normal comparisons.

N.B. Umbo results for the Malleal Rim might not be reliable as the grafting materials covered the umbo
