# Supplementary material for: Repair of subtotal tympanic membrane perforations: A temporal bone study of several tympanoplasty materials
Source: PLoS One. 2019 Sep 19;14(9):e0222728. doi: 10.1371/journal.pone.0222728 (PMC6752791; doi:10.1371/journal.pone.0222728)
Supplement: S4 Table — Summary of the significant differences between the different grafting materials and the normal TM stapes velocities for central perforation leaving Malleal Rim condition * = the mean difference is significant at the .0167 level for comparisons between graft conditions, and 0.00111 for graft-Normal comparisons. (DOCX) [file pone.0222728.s004.docx]

**S4 Table.** Summary of the significant differences between the different grafting materials and the normal TM stapes velocities for ***central perforation leaving Malleal Rim condition***

*= the mean difference is significant at the .0167 level for comparisons between graft conditions, and 0.00111 for graft-Normal comparisons.

| STAPES velocity | | Low Freq (250-500)  Mean dB difference (SE) | Middle Freq (1000-2000)  Mean dB difference (SE) | High Freq (3174-6349) Mean dB difference (SE) |
| --- | --- | --- | --- | --- |
| normal | thickCart | -9.943 (1.109) *  *p* < 0.0005 | -16.165 (.920) *  *p* < 0.0005 | -12.452 (.965) *  *p* < 0.0005 |
| normal | thinCart | -8.845 (1.265) *  *p* < 0.0005 | -14.374 (.999) *  *p* < 0.0005 | -13.818 (.855) *  *p* < 0.0005 |
| normal | silastic | -8.459 (1.381) *  *p* < 0.0005 | -11.149 (.892) *  *p* < 0.0005 | -13.328 (.890)*  *p* < 0.0005 |
| normal | Lotriderm | -13.111 (1.710) *  *p* < 0.0005 | -16.111 (.925) *  *p* < 0.0005 | -13.239 (.962) *  *p* < 0.0005 |
| normal | perichond | -6.059 (1.268) *  *p* < 0.0005 | -10.587 (.975) *  *p* < 0.0005 | -10.867 (1.138) *  *p* < 0.0005 |
| thickCart | thinCart | -1.0979 (1.926) | -1.791 (1.333) *  *p* =0.003 | 1.365 (1.368) |
| thickCart | silastic | -1.484 (1.926) | -5.016 (1.333) | .875 (1.368) |
| thickCart | Lotriderm | 3.167 (1.926) | -.0537 (1.333) *  *p* =0.001 | .786 (1.368) |
| thickCart | perichond | -3.883 (1.926) | -5.578 (1.333) | -1.584 (1.368) |
| thinCart | silastic | -.386 (1.926) | -3.224 (1.333) | -.490 (1.368) |
| thinCart | Lotriderm | 4.265 (1.926) | 1.737 (1.333) | -.579 (1.368) |
| thinCart | perichond | -2.785 (1.926) | -3.787 (1.333) | -2.950 (1.368) |
| silastic | Lotriderm | 4.652 (1.926) | 4.962 (1.333) *  *p* =0.003 | -.088 (1.368) |
| silastic | perichond | -2.399 (1.926) | -.562 (1.333) | -2.460 (1.368) |
| Lotriderm | perichond | -7.051 (1.926) *  *p* =0.004 | -5.524 (1.333) *  *p* =0.001 | -2.371 (1.368) |
