# Supplementary material for: Repair of subtotal tympanic membrane perforations: A temporal bone study of several tympanoplasty materials
Source: PLoS One. 2019 Sep 19;14(9):e0222728. doi: 10.1371/journal.pone.0222728 (PMC6752791; doi:10.1371/journal.pone.0222728)
Supplement: S5 Table — Summary of the significant differences between the different grafting materials and the normal TM umbo velocities for central perforation leaving Annular Rim condition * = the mean difference is significant at the .0167 level for comparisons between graft conditions, and 0.00111 for graft-Normal comparisons. (DOCX) [file pone.0222728.s005.docx]

**S5 Table.** Summary of the significant differences between the different grafting materials and the normal TM umbo velocities for ***central perforation leaving Annular Rim condition***

| UMBO velocity | | Low Freq (250-500)  Mean dB difference (SE) | Middle Freq (1000-2000)  Mean dB difference (SE) | High Freq (3174-6349)  Mean dB difference (SE) |
| --- | --- | --- | --- | --- |
| normal | thickCart | 6.153 (1.274) *  *p* < 0.0005 | -6.496 (1.444) *  *p* < 0.0005 | -13.925 (1.130) *  *p* < 0.0005 |
| normal | thinCart | 5.554 (1.376) *  *p* =0.001 | -6.943 (1.574) *  *p* < 0.0005 | -15.156 (1.000) *  *p* < 0.0005 |
| normal | silastic | 7.665 (1.287) *  *p* < 0.0005 | -1.097 (1.834) | -11.167 (1.342) *  *p* < 0.0005 |
| normal | Lotriderm | 5.177 (1.713) *  *p* =.006 | -12.373 (1.428) *  *p* < 0.0005 | -15.701 (.719) *  *p* < 0.0005 |
| normal | perichond | -2.708 (1.149) | -1.402 (1.312) | -11.137 (1.045) *  *p* < 0.0005 |
| thickCart | thinCart | .599 (1.942) | .447 (2.162) | 1.231 (1.508) |
| thickCart | silastic | -1.512 (1.942) | -5.398 (2.162) | -2.757 (1.508) |
| thickCart | Lotriderm | .976 (1.942) | 5.877 (2.162) | 1.776 (1.508) |
| thickCart | perichond | 8.861 (1.942)*  *p* < 0.0005 | -5.093 (2.162) | -2.787 (1.508) |
| thinCart | silastic | -2.111 (1.942) | -5.845 (2.162) | -3.989 (1.508) |
| thinCart | Lotriderm | .376 (1.942) | 5.430 (2.162) | .544 (1.508) |
| thinCart | perichond | 8.261 (1.942) *  *p* < 0.0005 | -5.540 (2.162) | -4.019 (1.508) |
| silastic | Lotriderm | 2.488 (1.942) | 11.276 (2.162) *  *p* < 0.0005 | 4.534 (1.508) |
| silastic | perichond | 10.373 (1.942) *  *p* < 0.0005 | .304 (2.162) | -.030 (1.508) |
| Lotriderm | perichond | 7.885 (1.942) *  *p* =0.001 | -10.971 (2.162) *  *p* < 0.0005 | -4.564 (1.508) |

*= the mean difference is significant at the .0167 level for comparisons between graft conditions, and 0.00111 for graft-Normal comparisons.
