# Supplementary material for: Repair of subtotal tympanic membrane perforations: A temporal bone study of several tympanoplasty materials
Source: PLoS One. 2019 Sep 19;14(9):e0222728. doi: 10.1371/journal.pone.0222728 (PMC6752791; doi:10.1371/journal.pone.0222728)
Supplement: S6 Table — Summary of the significant differences between the different grafting materials and the normal TM stapes velocities for central perforation leaving Annular Rim condition * = the mean difference is significant at the .0167 level for comparisons between graft conditions, and 0.00111 for graft-Normal comparisons. (DOCX) [file pone.0222728.s006.docx]

**S6 Table.** Summary of the significant differences between the different grafting materials and the normal TM stapes velocities for ***central perforation leaving Annular Rim condition***

*= the mean difference is significant at the .0167 level for comparisons between graft conditions, and 0.00111 for graft-Normal comparisons.

| STAPES velocity | | Low Freq (250-500)  Mean dB difference (SE) | Middle Freq (1000-2000)  Mean dB difference (SE) | High Freq (3174-6349)  Mean dB difference (SE) |
| --- | --- | --- | --- | --- |
| normal | thickCart | -2.650 (1.639) | -16.197 (1.043)*  *p* < 0.0005 | -15.683 (1.760)*  *p* < 0.0005 |
| normal | thinCart | 0.933 (1.398) | -14.917 (1.395)*  *p* < 0.0005 | -17.490 (1.549)*  *p* < 0.0005 |
| normal | silastic | -1.827 (1.388) | -12.408 (1.236)*  *p* < 0.0005 | -16.822 (1.632)*  *p* < 0.0005 |
| normal | Lotriderm | -7.777 (.986)*  *p* < 0.0005 | -23.126 (.815)*  *p* < 0.0005 | -19.386 (1.795)*  *p* < 0.0005 |
| normal | perichond | -5.919 (1.535)*  *p* =0.001 | -4.894 (1.213)*  *p* = 0.001 | -12.090 (1.318)*  *p* < 0.0005 |
| thickCart | thinCart | -1.716 (1.989) | 1.279 (1.636) | 1.806 (2.291) |
| thickCart | silastic | -.822 (1.989) | -3.789 (1.636) | 1.138 (2.291) |
| thickCart | Lotriderm | 5.127 (1.989) | 6.929 (1.636)*  *p* < 0.0005 | 3.702 (2.291) |
| thickCart | perichond | 3.269 (1.989) | -11.302 (1.636)*  *p* < 0.0005 | -3.593 (2.291) |
| thinCart | silastic | .893 (1.989) | -2.509 (1.636) | -.667 (2.291) |
| thinCart | Lotriderm | 6.843 (1.989)*  *p* =0.008 | 8.209 (1.636)*  *p* < 0.0005 | 1.896 (2.291) |
| thinCart | perichond | 4.986 (1.989) | -10.023 (1.636)*  *p* < 0.0005 | -5.399 (2.291) |
| silastic | Lotriderm | 5.949 (1.989) | 10.718 (1.636)*  *p* < 0.0005 | 2.563 (2.291) |
| silastic | perichond | 4.092 (1.989) | -7.513 (1.636)*  *p* < 0.0005 | -4.732 (2.291) |
| Lotriderm | perichond | -1.857 (1.989) | -18.232 (1.636)*  *p* < 0.0005 | -7.296 (2.291) |
